# Methods of Determining Energy Expenditure in Individuals with Prader-Willi Syndrome: A Systematic Literature Review

**DOI:** 10.3390/nu16132161

**Published:** 2024-07-07

**Authors:** Anela Halilagic, Danielle K. Longmore, Heather Gilbertson, George Moschonis

**Affiliations:** 1 Murdoch Children’s Research Institute, Parkville, VIC 3052, Australia; a.halilagic@latrobe.edu.au (A.H.); danielle.longmore@mcri.edu.au (D.K.L.); 2 The Royal Children’s Hospital, Parkville, VIC 3052, Australia; heather.gilbertson@rch.org.au; 3 Department of Food, Nutrition and Dietetics, School of Allied Health, Human Services and Sport, La Trobe University, Bundoora, VIC 3086, Australia

**Keywords:** Prader-Willi syndrome, obesity, nutritional intervention, energy requirements, resting energy expenditure, basal metabolic rate, predictive equation

## Abstract

Prader-Willi syndrome (PWS) is a rare disorder characterised by varying nutritional phases that occur throughout the lifespan, ranging from failure to thrive to hyperphagia. If uncontrolled, the imbalance between energy intake and expenditure results in obesity development and increased morbidity and mortality risk. Although measures of energy requirements for accurate nutrition assessment are vital, the evidence appears sparse and heterogeneous; hence, the aim of this review was to examine the available literature on energy expenditure predicted or measured using various methods in individuals with PWS. Studies were sought that presented methods and results on resting energy expenditure or basal metabolic rate. A narrative synthesis was completed to present the study characteristics and results. Methods of determining energy requirements included predictive equations and indirect calorimetry. Differences amongst ages, growth hormone therapy, fasting status, and measures in which results were presented were limitations to appropriately summarising and identifying trends in energy expenditure. Indirect calorimetry was identified as the most accurate method; however, it is not widely available in all settings. Further research is encouraged to support the development of valid and reliable predictive equations that will better inform and improve the efficiency of clinical practice in supporting people with PWS.

## 1. Introduction

Prader-Willi syndrome (PWS) is a rare genetic disorder with an incidence of 1 in 15,000–30,000 [1], originating from a defective paternal chromosome, 15q11.2-q13 [2]. It is characterised by hypotonia, developmental and cognitive delays, behavioural problems, sleep disorders, and neuroendocrine abnormalities [3]. Alongside this, different nutritional phases also occur throughout the lifespan of individuals with PWS [4].

Children with PWS present with hypotonia and weak sucking during early infancy, often resulting in nutritional deficiencies and failure to thrive. Appetite and feeding improvements occur later in infancy (9–24 months), where growth and weight gain then return to normal rates [4]. Couto-Rosende et al. (2023) [4] describe how, from two years of age, children with PWS begin to gradually develop an increased interest in food, which is associated with weight gain that continues throughout childhood. Hyperphagia and a lack of satiety then develop, which, if left uncontrolled, result in a chronic energy imbalance between energy intake and energy expenditure and are linked with the development of early childhood obesity [1]. Without preventative interventions, individuals with PWS are at greater risk of increased morbidity and mortality, since obesity development is highlighted as the most important health problem in this population [4]. The obesity associated with PWS differs from common obesity, where less lean body mass (LBM) and higher body fat (BF) are seen in those with PWS when compared with non-syndromic individuals matched for BMI [4].

Most individuals with PWS have growth hormone deficiency (GHD), which is associated with decreased LBM, increased fat mass (FM), hypotonia, low resting energy expenditure (REE), and decreased movement [4]. Further to this, total energy expenditure (TEE) is also lower because of additional factors, including decreased physical activity levels and lower energy utilisation related to reduced fat-free mass (FFM) [1,4].

Preventing obesity in individuals with PWS requires a combination of dietetic, physical, psychological, and behavioural interventions [4]. Current recommendations for nutrition intervention include restricting intake of energy (kJ or kcal) to prevent an energy imbalance, with varying values suggested ranging from 6 to 7 kcal (not specific to children or adults) [3] to 8–11 kcal for children per centimetre of height [4]. Other relevant recommendations suggest that the intake of children should be limited from 60% [5] to 60–80% [4] of the caloric total of a normal diet for a non-syndromic individual matched for height and age, with another recommendation of 60–75% intake, however not specific to children or adults [6]. In contrast, a study investigating the energy needs of children with PWS that underwent GHT found that children with PWS only required 3.2% fewer calories than healthy children to maintain growth [4].

**Figure 1:**
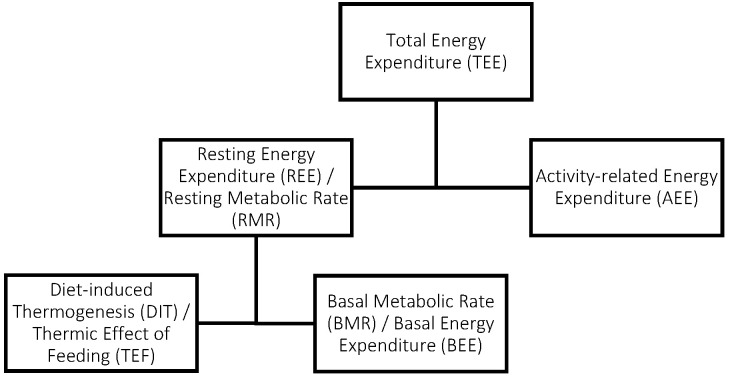
Components of energy expenditure [8].

In order to complete nutrition assessments and determine the effectiveness of interventions implemented for individuals with PWS, it is necessary to conduct accurate assessments of energy requirements [1]. Lazzer et al. (2016) have stated that basal metabolic rate (BMR) accounts for 70–80% of daily expenditure for PWS patients and therefore provides a basis for determining energy requirements. BMR is defined as the minimal energy required to maintain vital functions, while REE is defined as the energy required at rest [7], with the other components of energy expenditure presented in Figure 1. The most accurate method of measuring or determining BMR is indirect calorimetry; however, this can be both costly and impractical in most settings; hence, predictive equations are the second-best practical alternative developed and used to predict energy requirements [1].

### Objectives

The primary aim of this systematic literature review was to examine the evidence currently available in the literature on resting energy expenditure determined using various methods in individuals with PWS. We also aimed to analyse any differences in energy expenditure results between age groups and sex.

## 2. Materials and Methods

The protocol for this systematic literature review was published on PROSPERO on 30 December 2023 (registration number: CRD42023494703). This review was drafted according to the guidelines of the Preferred Reporting Items for Systematic Reviews and Meta-Analyses (PRISMA) statement [9].

### 2.1. Eligibility Criteria

This review sought studies that described methods of determining energy requirements in the PWS population, and that also presented subsequent results. The population group of interest was individuals with PWS aged from infancy (0 years) to adulthood (no age limit). There were no age and publication limitations placed due to the limited amount of available research on children with PWS. The primary outcomes of interest included measures of EE, such as Resting Energy Expenditure (REE), Resting Metabolic Rate (RMR), Basal Metabolic Rate (BMR), and Basal Energy Expenditure (BEE). Other potential additional outcomes included body weight, height, body composition values, and BMI to assess a correlation between anthropometric measures and energy requirements. Studies were eligible for inclusion if they were peer-reviewed, in English, and involved human participants. All study designs were included except for systematic literature reviews; however, reference lists were screened for any other eligible studies. Other exclusion criteria included if the outcomes of interest were not measured or reported and if components of EE other than resting or basal (i.e., Total Energy Expenditure) were reported only.

### 2.2. Searches and Sources

The database searches were conducted using Medline, PubMed, Cochrane, CINAHL, and Web of Science. Both published and non-published studies were sought. The date that the searches were last conducted on was the 2 of January 2024. The search strategy was based on the PCC framework proposed by the Joanna Briggs Institute [10], which includes population, concept, and context. This study was not limited to any context (i.e., geographical location or clinical setting); therefore, we avoided limiting our searches by not including this subheading. A template of the keywords and medical subject heading (MeSH) used to form the search strategies is identified in Table 1 below. Boolean operators were used with “AND” used, to combine terms within each PCC category and “OR” used to combine each category.

### 2.3. Study Selection

The initial database searches were completed by one reviewer, A.H., and then followed by exporting results to Endnote 21 and then Covidence. Endnote 21 was used to store the list of references, while Covidence was used to begin the title and abstract screening process. This initial screening was also completed by one reviewer, A.H., to exclude studies based on non-eligibility. Following this, the full-text screening process was conducted by two reviewers, A.H. and G.M., who independently used the inclusion criteria to determine the final number of studies to be included. One author was contacted with a request to provide additional results from their study [6]. Any disagreements were planned to be resolved by a third reviewer, H.G.; however, this was not required.

### 2.4. Data Extraction

The data extraction process began with one study completed by the first reviewer, A.H., and then confirmed by the second reviewer, G.M., to allow for discussion of any differences in opinion. The remainder of the included studies then underwent data extraction by the first reviewer, A.H. All extracted data was organised into a template in an Excel spreadsheet with the following variables as headings: title, authors, year of publication, study design, aim/objectives, country, setting/recruitment, study dates, inclusion/exclusion criteria, age, sample size, sex, sociodemographic characteristics, medical conditions/comorbidities, medication, method(s) of determining energy requirements, fasting/resting/test duration, comparison/control group, energy requirement measure, statistical analysis, and results. The selection of these variables was dictated by the relevant literature and the PCC framework, in addition to the development of the search strategy, which was also used to guide the development of the data extraction form.

Medical conditions and comorbidities included any diagnoses listed by the authors when describing the sample population in their study. It was important to identify any conditions other than PWS that could potentially have an influence on the EE results. Medication included any growth hormone therapy or treatment (GHT) that either occurred prior to or at the time of the study, as it has been reported that GHT is associated with beneficial effects on body composition [11], as well as increased RMR in children with PWS [12]. The method of determining energy requirements included any predictive equations or calorimetry used, while the energy requirement measure was looking for the specific measure used (i.e., REE, BMR) to allow for further synthesis and comparison.

The extracted data from studies was summarised into a table outlining the features most relevant to this review. This included the study design, location, age of sample, sample size, medical history, method of predicting or measuring energy requirements, energy measure, results, and quality appraisal findings. The order of the studies that are presented in the table was organised based on age, in ascending order, with three categories of ‘Children’, ‘Children and Adults’, and ‘Adults’.

### 2.5. Quality Appraisal

The JBI Critical Appraisal Tools [13] were used to assess the quality and risk of bias within each study. These tools are study design-specific; therefore, each study was assessed using the most appropriate tool. This was completed by one reviewer, A.H., where studies were scored based on the criteria, and then it was determined whether each study would be included, excluded, or if further information was required. Although various tools were used depending on the design of each study, some of the areas that were assessed included inclusion and exclusion criteria, study subjects, measurement of exposure and outcomes, identification of confounding factors or limitations, and follow-up. More specific to randomised controlled trials, allocation and blinding were also assessed where appropriate.

### 2.6. Data Synthesis

A narrative synthesis was conducted, which allowed for a summary to be developed of the results presented by each study. This involved identifying the similarities and differences between studies, assessing the methods used, outcome results, and highlighting the quality of each study. A meta-analysis was not appropriate at the time of this review due to insufficient homogeneity related to samples, methods, and results. Additionally, as there was no association or comparison being assessed due to the nature of the outcomes being descriptive (i.e., REE, BMR), a meta-analysis would have been inappropriate to conduct. As this review focused on a narrative method, the use of measures of consistency to determine heterogeneity between studies was not required.

## 3. Results

### 3.1. Study Selection

**Figure 2:**
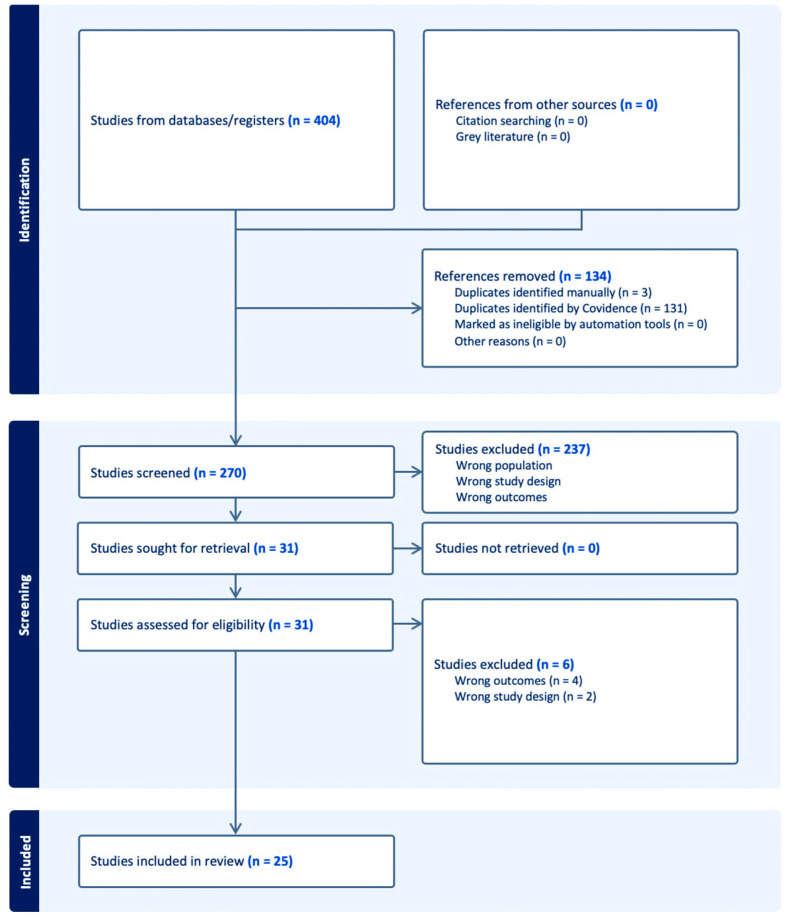
PRISMA flow diagram.

Following the initial searches, there were 404 identified studies. Following the removal of 134 duplicates in Covidence, there were 270 studies remaining to undergo title and abstract screening. It was identified that 237 studies were to be excluded during this screening process. Thirty-one studies then underwent full-text screening, where they were selected to either be included or excluded based on eligibility criteria, resulting in a further six studies being excluded due to the wrong outcome or study design (Figure 2). The final number of studies selected for this review was 25, including 14 cross-sectional, one longitudinal cohort, eight randomised controlled, and two quasi-experimental studies.

### 3.2. Study Characteristics

**Figure 3:**
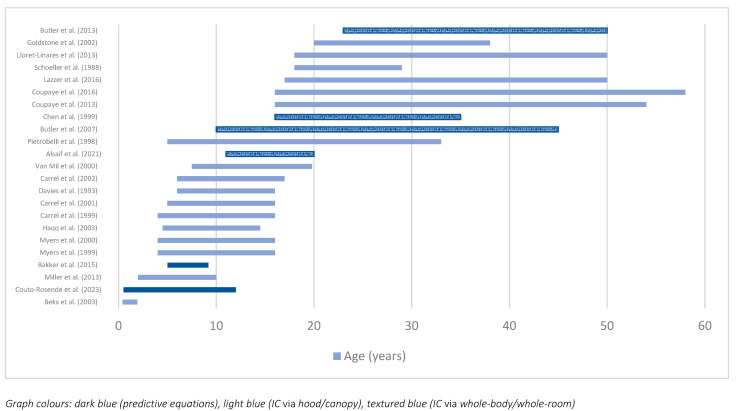
Age ranges and methods of predicting or measuring energy expenditure [1,3,4,5,9,12,13,15,16,17,18,19,20,21,22,24,25,26,27,28,29,30,31].

The locations of the studies ranged across various countries; however, the majority (*n* = 13) were located in the USA. One of these studies also included participants from Canada [3]. The remaining locations included three studies in France [11,14,15], three in Italy [1,2,16], two in the UK [17,18], two in the Netherlands [19,20], one in Canada [21], and one in Spain [4]. The target population in 12 studies was children; six studies examined adults; and seven studies were conducted with both children and adults, with the youngest and oldest age included in the studies selected for this review being 5 months [22] and 58 years [14], respectively. Figure 3 presents the age range of each of the study samples, with the smallest range being 18 months [22] and the largest range being 42 years [14]. Two studies [2,23] were excluded from this graph (Figure 3) due to the age of their samples being presented in mean +/− standard errors with no range available. Furthermore, there was a wide variation in sample sizes within the selected studies, with the smallest sample including only 2 [24] and the largest one including 89 [2] participants.

### 3.3. Methods of Predicting or Determining Energy Requirements

The different methods that were used to predict or measure energy requirements in the studies are presented below (Table 2). Studies by Couto-Rosende et al. (2023) [4] and Bakker et al. (2015) [19] both used predictive equations, the Henry equation and Muller’s equation, respectively. The vast majority of studies (*n* = 19) used the hooded or canopy method of indirect calorimetry to measure the energy requirements of their PWS participants. The remainder of studies (*n* = 4) used whole-body or whole-room calorimetry [21,24,25,26]. Some of these studies (*n* = 10) also reported the equation used for calculating energy expenditure by inputting VO_2_ and CO_2_ determined via indirect calorimetry. These equations included the Fleisch equation, which was used within all three studies by Carrel et al. [27,28,29], the Jequier et al. equation, which was used within one study by Butler et al. (2013) [25], and the remaining six studies used the Weir equation [1,2,11,20,24,26]. The methods have also been displayed in Figure 3 via the colouring of the bar graph; dark blue represents predictive equations, light blue represents indirect calorimetry via canopy or hood, and textured blue represents whole-room or whole-body indirect calorimetry.

The fasting status of participants undergoing indirect calorimetry, both via the canopy or hood and whole-room methods, was reported by 18 studies, with only one of these not specifying the duration [2]. The remaining included a 6-h fast in one study [22], an 8-h fast in three studies [12,21,23], a 12-h fast in six studies [16,27,28,29,30,31], and an overnight fast in seven studies [1,3,15,18,20,24,26]. One study by Butler et al. (2013) [25] reported that fasting status could not be determined, and the remaining four studies did not report fasting status [5,11,14,17].

**Table 2:** Characteristics and data extraction of included studies (*n* = 25).

		Sample						
Study		Location and Age	*n*	Medical	Method	Energy Measure	Results	Quality Appraisal
Children	[22]	USA 5–23 months	14	PWS diagnosis	Indirect calorimetry, 6-h fast	REE	REE (kcal/day) 758 +/− 477	Overall: Include - Inclusion/exclusion criteria not clear
	[4]	Spain 6 months-12 years	25	PWS diagnosis, GHT: 25 at the time of study	Henry equation [32]	BMR	BMR (kcal/day) 1290 +/− 317	Overall: Include
	[3]	USA and Canada 2–10 years	63	PWS diagnosis, GHT: 61 at time of study	Indirect calorimetry, overnight fast	REE	REE (kJ/day) Energy restricted diet: 4668.9 kJ (=1117.0 kcal) Energy restricted and modified composition: 3935.1 kJ (=941.4 kcal)	Overall: Include
	[19]	Netherlands 5.0–9.2 years	47	PWS diagnosis	Muller’s equation: REE (kcal/day) = (0.0788 × FFM (kg) + 0.02132 × FM (kg) + 0.327 × gender + 2.694) × 1000/4.18	REE	REE (kcal/day) Baseline: 899 (865–991) After 2 years GHT: 971 (948–1151) After 2 years control group: 1023 (962–1095)	Overall: Include - Blindness to group assignment not clear
	[30]	USA 4–16 years	54	PWS	Indirect calorimetry, 12-h fast	REE	REE (kcal/m^2^/h) Baseline: 22.5 +/− 3.4 (control), 22.4 +/− 4.4 (treatment) After 12 months GHT: 25.1 +/− 6.9 (control group), 28.2 +/− 7.4 (GHT group)	Overall: Include - Blindness to group assignment not clear
	[31]	USA 4–16 years	16	PWS	Indirect calorimetry, 12-h fast	REE	REE (kcal/m^2^/h) Baseline: 22.5 +/− 3.5 (control), 22.4 +/− 4.4 (treatment) After 12 months GHT: 25.1 +/− 7.0 (control), 28.3 +/− 7.5 (treatment) After 24 months GHT: 29.2 +/− 7.8	Overall: Include - Blindness to group assignment not clear
	[12]	USA 4.5–14.5 years	14	PWS diagnosis, GHT: None prior to study	Indirect calorimetry, 8-h fast	REE	REE (kcal/day) Baseline: 1288 +/− 290 After 6 months placebo: 1285 +/− 404 After 6 months GHT: 1533 +/− 455	Overall: Include
	[23]	USA 11.7 +/− 4.6 years	4	PWS diagnosis, GHT: 2 at the time of study	Indirect calorimetry, 8-h fast	REE	REE (kcal/day) Baseline: 1824 +/− 338.5 After 5–7 days GHT: 1720.8 +/− 186.3	Overall: Include
	[27]	USA 4–16 years	54	PWS diagnosis, GHT: None prior to study	Indirect calorimetry, 12-h fast, Fleisch equation	REE	REE (kcal/m^2^/h) Baseline: 22.5 +/− 3.4 (control), 22.4 +/− 4.4 (treatment) After 12 months GHT: 25.1 +/− 6.9 (control), 28.2 +/− 7.4 (treatment)	Overall: Include - Blindness to group assignment not clear
	[28]	USA 5–16 years	26	PWS diagnosis GHT: 26 in previous RCT	Indirect calorimetry, 12-h fast, Fleisch equation: Men: 54.337821 – (1.19961 × Age) + (0.02548 × Age^2^) – (0.00018 × Age^3^), Women: 54.74942 – (1.54884 × Age) + (0.03580 × Age^2^) – (0.00026 × Age^3^)	REE	REE (kcal/m^2^/h) Baseline: 22.5 +/− 3.8 After 24 months GHT (1.0 mg/m^2^): 29.8 +/− 7.5 After additional GHT 24–36 months (1.5 mg/m^2^): 29.2 +/− 7.9 After additional GHT 24–36 months (1.0 mg/m^2^): 29.8 +/− 4.2 After additional GHT 24–36 months (0.3 mg/m^2^): 26.4 +/− 5.2	Overall: Include - Blindness to group assignment not clear
	[17]	UK 6–16 years	10	PWS diagnosis	Indirect calorimetry	RMR	RMR (kcal/day) 1323 +/− 421	Overall: Include - Inclusion/exclusion criteria not clear
	[29]	USA 6–17 years	26	PWS diagnosis, GHT: 26 in previous RCT	Indirect calorimetry, 12-h fast, Fleisch equation	REE	REE (kcal/m^2^/h) Baseline: 22.5 +/− 4.2 After 24 months GHT: 29.0 +/− 6.2 After additional GHT 24–48 months (1.5 mg/m^2^): 35.3 +/− 5.9 After additional GHT 24–48 months (1.0 mg/m^2^): 32.9 +/− 6.7 After additional GHT 24–48 months (0.3 mg/m^2^): 30.3 +/− 5.1	Overall: Include - Blindness to group assignment not clear
Children and Adults	[20]	Netherlands 7.5–19.8 years	17	PWS GHT: None	Indirect calorimetry, overnight fast, Weir’s equation: REE = (3.94 × VO2) + (1.1 × VCO2)	BMR	BMR (MJ/day): 5.36 MJ +/− 1.18 (=1282 kcal) BMR (MJ/day) adjusted for weight: 5.17 MJ +/− 1.57 (=1237 kcal) BMR (MJ/day) adjusted for FFM: 5.31 MJ +/− 1.38 (=1270 kcal)	Overall: Include - Inclusion/exclusion criteria not clear
	[21]	Canada 11–20 years	5	PWS diagnosis, GHT: 5 at the time of study	Open-circuit whole-body calorimetry unit (WBCU), 8-h fast	REE	REE (kcal/day) 1686 +/− 234	Overall: Include - Not blinded
	[16]	Italy 5–33 years	21	PWS	Indirect calorimetry, 12 h fast	REE	REE (kcal/day) 1719.5 +/− 478	Overall: Include - Inclusion/exclusion criteria not clear
	[26]	USA 10–45 years	48	PWS diagnosis, GHT: None	Whole-room indirect calorimeter, overnight fast, Weir equation	REE	REE (kcal/min) Total sample: 1.44 +/− 0.25 (=2074 kcal/day) Male: 1.53 +/− 0.24 (=2203 kcal/day) Female: 1.37 +/− 0.23 (=1973 kcal/day)	Overall: Include - Inclusion/exclusion criteria not clear
	[24]	USA 16–35 years	2	PWS diagnosis	Whole-room indirect calorimeter, overnight fast, Weir equation	REE	REE (kJ/min) Male: 5.74 kJ (=1.37 kcal/min or 1973 kcal/day) Female: 3.63 kJ (=0.87 kcal/min or 1253 kcal/day)	Overall: Include - Inclusion/exclusion criteria not clear
	[11]	France 16–54 years	40	PWS diagnosis, GHT: 11 prior to study, 0 at the time of study	Indirect calorimetry, Weir equation	RMR	RMR (kcal/day) Total sample: 1672 +/− 405 Sub-sample that received GHT during childhood: 1702 +/− 384 Sub-sample that did not receive GHT during childhood: 1593 +/− 264	Overall: Include
	[14]	France 16–58 years	57	PWS diagnosis	Indirect calorimetry	REE	REE (kcal/day) Deletion group: 1746 +/− 367 UPD group: 1600 +/− 422	Overall: Include
Adults	[1]	Italy 17–50 years	80	PWS diagnosis, GHT: 29 prior to study, 17 at the time of study	Indirect calorimetry, overnight fast, Weir’s equation	BMR	BMR (MJ/day): male, female Group 1: 7.68 MJ +/− 1.41 (= 1837 kcal), 6.40 MJ +/− 1.51 (=1531 kcal) Group 2: 7.62 MJ +/− 1.60 (= 1822 kcal), 5.91 MJ +/− 1.50 (=1414 kcal) BMR (MJ/day) – adjusted for FFM and FM Group 1: 7.34 MJ +/− 1.25 (=1756 kcal), 6.67 MJ +/− 1.32 (=1596 kcal) Group 2: 7.21 MJ +/− 1.42 (=1725 kcal), 6.23 MJ +/− 1.23 (=1490 kcal) New equations developed: BMR = BM × 0.052 + sex × 0.778 - age × 0.033 + 2.839 (anthropometric) BMR = FFM × 0.074 + FM × 0.042 + sex × 0.636 - age × 0.037 (body comp.)	Overall: Include - Inclusion/exclusion criteria not clear
	[5]	USA 18–29 years	6	PWS diagnosis	Indirect calorimetry	BMR	BMR (kcal/day): 1160 +/− 95 Male: 1187 Female: 1133	Overall: Include - Inclusion/exclusion criteria not clear
	[15]	France 18–50 years	27	PWS diagnosis, GHT: 5 prior to study, 1 at the time of study	Indirect calorimetry, overnight fast	BMR	BMR (kcal/day) Male: 1946 +/− 428 Female: 1758 +/− 360	Overall: Include
	[33]	Italy 28.4 +/− 8.7 years	89	PWS diagnosis, GHT: 22 at the time of study	Indirect calorimetry, fasting (duration not specified), Weir’s equation	REE	REE (kcal/day) 1455 +/− 310	Overall: Include
	[18]	UK 20–38 years	8	PWS diagnosis, GHT: None	Indirect calorimetry, overnight fast	RMR	RMR (MJ/day) 6.64 MJ +/− 0.46 (=1588 kcal)	Overall: Include - Inclusion/exclusion criteria not clear
	[25]	USA 23–50 years	11	PWS diagnosis	Whole-room indirect calorimetry, fasting status could not be determined, Jequier et al. equation	RMR	RMR (kcal/day): mean, SE Baseline: 1845, 161 After 12 months GHT: 1919, 203 After cessation 12–24 months: 1914, 278	Overall: Include

### 3.4. Energy Requirement Measures

The components of EE are depicted in Figure 1, which also shows the relevant synonyms. The energy components that were measured in the studies included in this review were REE, RMR, and BMR. REE/RMR was the outcome measure in 20 studies, while the remaining five studies measured BMR [1,4,5,15,20]. The units that energy expenditure (i.e., as REE/RMR, BMR) is measured also vary between studies (Table 2), with 20 studies using kilocalories (kcal), two studies using kilojoules (kJ) [3,24], and three studies using megajoules (MJ) [1,18,20]. Additionally, results varied in regard to whether energy expenditure was presented per day, per minute [24,26], or per m^2^/h [27,28,29,30,31], with m^2^ being based on body surface area. Where possible, calculations were made in order to present all energy expenditure units in kcal/day, for ease of result comparison. Kcal/day was selected as this was the unit that most studies had originally presented their results as.

### 3.5. Growth Hormone Therapy

Growth hormone therapy is an approved treatment for children with PWS, as it has been shown to increase growth rate and decrease FM, with some studies also reporting an increase in resting metabolic rate [11]. Growth hormone therapy or treatment (GHT) was included in the data extraction process and can also be found in the ‘Medical’ column in Table 2. Ten studies did not report any information about GHT occurring [5,14,16,17,19,22,24,25,30,31], while only three studies actually reported that there was no GHT prior to or at the time of the study [18,20,26]. There were nine studies that included GHT as part of the treatment, which can be identified in the ‘Results’ column of Table 2, where any results that occurred prior to or following GHT have been identified accordingly [12,19,23,25,27,28,29,30,31]. Two of these [12,27] reported that there was no GHT prior to the studies occurring, while four did not mention if there was any GHT prior to study commencement [19,25,30,31]. The specifics of GHT medication and dosage were not included in data extraction and synthesis unless they were directly related to energy expenditure results [28,29].

### 3.6. Studies on Children

Energy expenditure generally increases as children age and throughout puberty, then plateaus in adulthood up until late adulthood in the sixth decade of life, where it begins to decline [34]. Studies that measured the energy expenditure of children with PWS are divided into two groups: one group includes studies with children only, and a second group includes studies where children and adults were combined. The studies included in the latter did not present any age subgroups to separate children and adults; therefore, their results are a mean of all ages.

There were two studies that included infants, one of which was infants only (5–23 months) [22], while the other additionally included school-aged children (6 months–12 years) [4], with a REE of 758 +/− 477 kcal/day and a BMR of 1290 +/− 317 kcal/day, respectively. Miller et al. (2013) [3] measured the REE of PWS children aged 2–10 years who were either on an energy-restricted diet or an energy-restricted combined with a modified composition diet (30% fat, 45% carbohydrates, and 25% protein) and found values of 1117.0 kcal/day and 941.4 kcal/day, respectively. Another study that examined similarly aged children (5–9.2 years) measured a baseline REE of 899 kcal/day and then completed measurements after two years of GHT (971 kcal/day) and a control group (1023 kcal/day).

Eight studies had population groups that included children from both school-aged and adolescent years, ranging from 4 to 17, and none were separated into age subgroups. Seven of these investigated the changes in REE following GHT, with differences in the units the results were presented in. Baseline values varied from 22.4 +/− 4.4 kcal/m^2^/h [30] to 1824 +/− 338.5 kcal/day [23]. Six out of the seven studies presented results that suggested an increase in REE following GHT; some results showed increases from 22.4 +/− 4.4 to 28.2 +/− 7.4 kcal/m^2^/h following 12 months [30], and to 29.2 +/− 7.8 kcal/m^2^/h following 24 months [31], and from 1288 +/− 290 to 1533 +/− 455 kcal/day following 6 months [12] of GHT. However, in their short-term study, Haqq et al. (2003) [23] measured REE after 5–7 days of GHT and found a decrease from 1824 +/− 338.5 to 1720.8 +/− 186.3 kcal/day.

The seven studies that included both children and adults in their samples presented the following results: BMR: 1282 kcal/day (7.5–19.8 years) [20], REE: 1686 +/− 234 kcal/day (11–20 years) [21], REE: 1719.5 +/− 478 kcal/day (5–33 years) [16], REE: 2074 kcal/day (10–45 years) [26], REE: 1973 kcal/day in males, 1253 kcal/day in females (16–35 years) [24], RMR: 1672 +/− 405 kcal/day (16–54 years) [11], and REE: 1746 +/− 367 kcal/day (PWS subtype: deletion), 1600 +/− 422 kcal/day (PWS subtype: UPD) (16–58 years) [14].

### 3.7. Studies on Adults

Lazzer et al. (2016) [1] measured BMR in two groups of adults aged 17–50 years with PWS and then developed two new predictive equations based on anthropometric and body composition values. The new equations developed were BMR = BM × 0.052 + sex × 0.778 − age × 0.033 + 2.839 (anthropometric using body mass) and BMR = FFM × 0.074 + FM × 0.042 + sex × 0.636 − age × 0.037 (body composition using FFM and FM). The BMR values were separated into male and female groups: Group 1: 1837 kcal/day (male), 1532 kcal/day (female), Group 2: 1822 kcal/day (male), and 1414 kcal/day (female). Groups 1 and 2 refer to two groups that the PWS sample was divided into: a calibration group, which was used to develop the new equations, and a validation group, which was used to validate the new equations.

There were five other studies that measured energy expenditure in adults only, with the ages ranging from 18 to 50 years. Baseline values for each of the studies were BMR: 1160 +/− 95 kcal/day (18–29 years) [5], BMR: 1946 +/− 428 (male), 1758 +/− 360 (female) kcal/day [15], REE: 1455 +/− 310 kcal/day (28.4 +/− 8.7 years) [2], RMR: 1588 kcal/day (20–38 years) [18], and RMR: 1845 (SE: 161) kcal/day (23–50 years) [25].

### 3.8. Sex Differences

There were a total of five studies that separated their energy expenditure results into male and female categories. In addition to the studies and results presented above by Lazzer et al. (2016) [1] and Lloret-Linares et al. (2013) [15], there were a further three studies. Butler et al. (2007) [26] and Chen et al. (1999) [24] measured both children and adults (combined) and presented the following: REE: 2203 kcal/day (male), 1973 kcal/day (female) [26], REE: 1973 kcal/day (male), 1253 kcal/day (female) [24]. Schoeller et al. (1988) [5] examined a study population aged 18–29 years and reported BMR values of 1187 kcal/day (male) and 1133 kcal/day (female).

### 3.9. Quality Appraisal

Following assessment of the quality of each study using the JBI tools, all 25 studies were included for synthesis and interpretation. A total of 14 studies were assessed using the checklist for analytical cross-sectional studies, with the main source of potential risk of bias being missing clear inclusion and exclusion criteria. Two studies underwent appraisal using the checklist for quasi-experimental studies, which resulted in the identification of the main sources of potential bias risk sourcing from missing control groups and missing or unclear information about multiple measurements being taken pre- and post-intervention. The checklist for randomised controlled trials was used for eight studies and found that unclearness about concealment of group allocation, blindness to treatment assignment, and blindness of those delivering treatment were the main potential risks of bias. The final study to undergo a quality appraisal was a cohort study where there were no sources of risk identified. The quality assessment results and sources of bias for individual studies are presented in Table 2.

## 4. Discussion

The age range of study participants in the selected studies (Figure 3) demonstrates a wide and even spread across the lifespan, ranging from infancy to mid-adulthood. This has allowed results to be presented and interpreted as an overview of the lifespan, while also exploring and highlighting any potential differences and comparisons that could be made between ages. The 25 studies included in this review all predicted or measured resting or basal energy expenditure in individuals with PWS. The characteristics of the populations varied in age, gender, and location, with further contrasts in fasting status, testing methods, and even potential comorbidities. The countries that the studies were conducted in included the USA, Canada, the United Kingdom, Spain, France, the Netherlands, and Italy, all of which are considered high-income countries [35]. This may be due to the reported disparities in scientific capacity between countries based on health researchers (in full-time equivalent) per million inhabitants by income group in different countries [36]. This will be later discussed alongside the accessibility and feasibility of equipment and the expertise of indirect calorimetry. Approximately one-third of the studies included in this analysis were randomised controlled studies, which provide the highest level of evidence after systematic literature reviews, while the remainder fall under quasi-experimental and observational studies, which represent the next two levels of evidence [37]. The combination of these study designs provides overall strength and quality to the evidence in the current systematic literature review.

### 4.1. Methods of Measuring

REE and BMR values have been measured and predicted using different modes of indirect calorimetry and predictive equations. Indirect calorimetry measures gas exchange, whereas direct calorimetry measures heat production [38]. The studies that conducted whole-room and whole-body calorimetry measured gas exchange [21,24,25,26], therefore these have been classified as indirect calorimetry. BMR is defined as the minimal energy required to maintain vital functions, while REE is defined as the energy required at rest [7]. REE is generally higher than BMR due to the inclusion of diet-induced thermogenesis (Figure 1), a measure of energy expended through digestion, absorption, and storage of nutrients [21]. Therefore any measurements of BMR require the individuals to be in a fasting state. Diet-induced thermogenesis contributes to approximately 10–15% of an individual’s TEE [21]. Not all studies included in this review that measured BMR had their sample group fasted for testing; in contrast, some studies that measured REE did. Fasting in this population group is inherently difficult due to their regimented diet intake related to controlling hyperphagia, as well as associated impulsive food behaviours such as stealing food [33]. This inconsistency in fasting status has the potential to impact the accuracy and consistency of the results reported in the current review.

### 4.2. Growth Hormone Therapy

Comparisons can be made between various results within individual studies, such as baseline and post-intervention measurements. Couto-Rosende et al. (2023) [4] describe how GHT is effective in increasing lean mass, improving muscle tone, and improving energy expenditure at rest. Several studies in this review demonstrate this association between GHT and improved REE [12,27,30,31]. Two further studies presented results that suggested a positive association between REE and GHT dosage, where higher dosages were correlated with greater improvements in energy expenditure [28,29]. However, the present review also identified two studies whose results did not support the above association: Bakker et al. (2015) [19] reported that REE was lower in the GHT group than the untreated group compared to baseline (using Muller’s equation), and Haqq et al. (2003) [12] reported results that demonstrated a reduced REE following 5–7 days of GHT.

### 4.3. Predictive Equations

The predictive equations used by Couto-Rosende et al. (2023) [4] and Bakker et al. (2015) [19] were the Henry equation [32] and Muller’s equation [39], respectively. Neither of these equations is specific to the PWS population; therefore it would potentially be at risk of overestimating energy expenditure in this group. Lazzer et al. (2016) developed new predictive equations for adults with PWS based on anthropometric and body composition measures. The new equations showed significantly higher accuracy for energy expenditure compared with most other predictive equations tested [1]. Compared to Henry and Muller’s equations, Lazzer’s equations are based on a cohort of adults with PWS, which would take into consideration the low LBM and high FM and their effect on an individual’s energy expenditure. The mean predicted BMR values estimated with the two new equations were not significantly different from the mean measured BMR (6.99 ± 1.37 and 6.92 ± 1.33 vs. 6.76 ± 1.74 MJ per day, respectively). The correlation coefficients between predicted BMR and measured BMR were R^2^ = 0.74 for equation 1 and R^2^ = 0.78 for Equation (2).

### 4.4. Factors Impacting REE and BMR

There were several factors identified in the analysis of this review that could have a potential impact on the results reported for REE and BMR. These include age, sex, GHT, fasting status, diet composition, the method of measurement, and even the formulas used in the background of IC. Measures of VO_2_ and CO_2_ taken by the IC are inputted into equations or formulas to calculate REE or BMR. Various equations were used amongst the studies in this review, resulting in potential differences in EE results. Several studies in this review separated their findings based on sex, allowing the reader to observe the difference in BMR and REE between males and females [1,5,15,24,25]. There were consistent findings of results being greater in males than females, ranging from a small difference of 54 kcal/day [5] to the largest difference of 720 kcal/day [24]. Age also has an important role in EE, as it would be expected that results would vary depending on the different stages of childhood and adulthood. The studies in this review demonstrate how the younger cohorts are associated with lower values of REE and BMR compared to their older counterparts. These variances highlight the value of presenting results as subgroups of age and sex so that important differences are understood. As discussed earlier, GHT has been found to improve EE at rest [4], which stresses the significance of reporting how many, if any, individuals in a sample group had taken part in GHT prior to or at the time of the study. There were several studies that had overlooked this information in their reporting and were missing data on GHT. These factors emphasise the importance of standardising methods so that the impact of confounding factors may be reduced when possible and consistent measures and results are obtained.

### 4.5. Strengths and Limitations

This review holds strength in the large number of studies identified to be included and analysed. This review used the recommended standard methods according to the PRISMA statement for systematic literature reviews. However, there were limitations, hurdles, and difficulties in interpreting the evidence from the selected studies. The evidence presents results that show REE and BMR values for infants, children, and adults, with some studies presenting results combining both children and adults. Variations are also seen in units (i.e., kcal, kJ, MJ), durations (i.e., /min, /day), and some studies even present their results as kcal/m^2^/h. The use of m^2^ limits the ability to compare these results to others due to the requirement of inputting individual anthropometric data. Large ranges of ages and the combination of different life stages in results also limit the ability for direct age-specific comparisons. This is further hindered by the lack of use of subgroups for ages, which would permit improved comparability in analysis. The identification of comorbidities amongst the populations is missing, which is a limitation of this study due to the potential impact on energy requirements.

Another potential limitation is publication bias; this may have been reduced if any unpublished studies were included; however, no unpublished studies were identified or selected in this review. Although a meta-analysis would have improved the quality of results, the nature of the identified studies and outcomes of interest (i.e., energy expenditure) values, along with the high level of heterogeneity amongst studies, did not justify a meta-analysis being completed. Alternatively, the complete set of studies was presented in Table 2 to provide an overview of the wide range of methods and findings while additionally being narratively summarised.

### 4.6. Implications and Recommendations

Our findings align with previously published studies that have highlighted the importance of accurately measuring EE in individuals with PWS as an integral part of dietary management. Obesity has been highlighted as the central health problem, resulting in a greater risk of increased morbidity and mortality for this population. We identified that IC, as the gold standard, was the only method that took body composition into consideration, which is vital for this population group. Although it is the most accurate method, IC is costly and not feasible in most clinical settings [1]. As detailed earlier, the countries that the studies in this review were conducted in were all high-income countries, likely increasing the likelihood of IC availability. Even within high-income countries, regional and rural areas are also generally limited in their access to clinical equipment and resources. For this reason, predictive equations are the next-best practical alternative used to predict energy requirements. Novel predictive equations by Lazzer et al. (2016) [1] were developed for adults with PWS due to an identified gap in research. The Henry equation and Muller’s equation identified in this review were used for children with PWS; however, we highlighted that both are based on the general population and therefore likely problematic for use in children with PWS. A systematic review published in 2017 that aimed to determine the presence of alterations in energy expenditure in individuals with PWS concluded that determining energy requirements by taking body composition into consideration is vital in the dietary management of this population [40]. The current predictive equations being used to assess REE incorporate the use of body weight and assume a “healthy” body composition, which was stated to be problematic in this cohort [21].

There is a gap in the current predictive equations that are available and PWS-specific, as there are no equations that have been developed for and targeted at the paediatric population. Future research should aim to fill this research gap while ensuring that the limitations identified in this study are taken into consideration to improve ease of interpretation and analysis of results.

## 5. Conclusions

The various methods of measuring EE components, BMR and REE, have been clearly identified among the studies in this review. Although there are ranging results dependent on several factors such as age, sex, and method of measurement used, this heterogeneity between included studies resulted in difficulty appropriately summarising and identifying trends in EE due to various limitations. IC was the singular method used that took into consideration the altered body composition of individuals with PWS; therefore, is identified as the most accurate and necessary method for this population. However, as IC is not widely available in all settings where individuals with PWS are monitored, further research is encouraged to support the development of practical, accurate, and cost-effective predictive equations of EE that will better inform and improve the efficiency of clinical practice.

## Figures and Tables

**Table 1:** Database search strategy example.

		Headings/Keywords	Term Type
Population	1.	Prader-Willi syndrome	MeSH
	2.	Prader-Willi syndrome	Keyword
	3.	1 or 2	
Concept	4.	Energy metabolism	MeSH
	5.	Basal metabolism	MeSH
	6.	Predictive equation	Keyword
	7.	Energy calculation *	Keyword
	8.	Nutritional requirements	MeSH
	9.	Energy requirement	Keyword
	10.	Calorimetry	MeSH
	11.	Calorimet *	Keyword
	12.	Resting energy expenditure	Keyword
	13.	Basal metabolic rate	Keyword
	14.	Resting metabolic rate	Keyword
	15.	Basal energy expenditure	Keyword
	16.	4 or 5 or 6 OR 7 or 8 or 9 or 10 or 11 or 12 or 13 or 14 or 15	
Context		-	
	17.	3 and 16	

Truncation symbol (*) used to find different word endings

## Data Availability

No new data were created or analysed in this study. Data sharing is not applicable to this article.
